# FOXO1-mediated argininosuccinate lyase transcription inhibits ammonia metabolism and breast cancer cell metastasis

**DOI:** 10.1016/j.jbc.2025.110677

**Published:** 2025-09-02

**Authors:** Min Zhao, Dongdong Yuan, Mengmeng Wei, Jie Zhang, Wenjing Yang, Shaojie Qin, Le Li

**Affiliations:** 1 School of Life Sciences, Ningxia University, Yinchuan, China; 2 General Hospital of Ningxia Medical University, Yinchuan, China

**Keywords:** FOXO1, ASL, arginine, urea cycle, breast cancer metastasis

## Abstract

Cancer cells exhibit altered and elevated metabolic processes to meet their increased bioenergetic and biosynthetic demands, leading to the production of ammonia as a byproduct. However, the mechanisms by which tumor cells manage excess ammonia remain poorly understood, despite its critical role in nitrogen metabolism. The urea cycle (UC), a central pathway for ammonia detoxification, has been insufficiently explored in the context of cancer metabolism. In this study, we identify Forkhead box O1 (FOXO1), a transcription factor essential for tumorigenesis and progression, as a key regulator of the UC in breast cancer cells. Specifically, FOXO1 inhibits argininosuccinate lyase (ASL) expression, a crucial enzyme in the UC, leading to reduced ammonia detoxification. Mechanistic analyses reveal that ASL is a direct transcriptional target of FOXO1. Functionally, we demonstrate that FOXO1 modulates the migratory ability of breast cancer cells through the regulation of ASL and arginine metabolism. These findings unveil an unexpected role of FOXO1 in regulating the UC in tumors and highlight a novel mechanism by which breast cancer cells exploit metabolic pathways to support their progression and metastasis. Our study provides valuable insights into cancer metabolism and identifies potential targets for therapeutic intervention.

Tumor initiation and progression rely on profound alterations in cellular metabolism to support increased demands for energy and biosynthesis, essential for rapid cancer cell proliferation (1, 2, 3). Aberrant metabolic activities in tumors frequently result in ammonia generation, a metabolic byproduct whose accumulation must be managed to maintain cellular homeostasis (4, 5, 6, 7). The urea cycle (UC), a central component of nitrogen metabolism, plays a critical role in detoxifying excess ammonia and maintaining nitrogen balance. Increasing evidence has demonstrated that tumors reprogram the UC to balance nitrogen waste while supporting anabolic demands. Our prior research demonstrated that upregulation of the UC plays an essential role in facilitating both ammonia clearance and cellular proliferation in tumor cells (8). Notably, recent work has shown that suppression of the UC enzyme ASS1 promotes tumor proliferation by diverting aspartate flux toward *de novo* pyrimidine biosynthesis, thereby underscoring the broader oncogenic implications of UC remodeling in cancer metabolism (9).

Argininosuccinate lyase (ASL), a key enzyme in the UC, catalyzes the conversion of argininosuccinate into arginine and fumarate—precursors of polyamines that promote cancer cell proliferation (10, 11). The abnormal expression patterns of ASL have been closely associated with poor prognoses in colorectal cancer, hepatocellular carcinoma, and breast cancer (12, 13). In our study, analyses of clinical datasets using online tools revealed that ASL expression is significantly elevated in metastatic breast cancer samples, supporting the relevance of breast cancer as a model to study ASL regulation. In addition, arginine deprivation has been shown to inhibit migration and invasion of colorectal cancer cells, highlighting the link between nitrogen metabolism and tumor progression (14, 15). While the role of ASL has been explored in several cancers, the mechanisms regulating its expression, particularly in breast cancer, remain poorly understood. Given the critical role of ASL in both metabolic flux and metastatic progression, elucidating its transcriptional regulation in breast cancer could uncover novel therapeutic targets. Metabolic pathway alterations in tumor cells are strongly correlated with aberrations in specific oncogenes or tumor suppressor genes (16).

The Forkhead box class O (FOXO) protein family is a highly conserved set of transcription factors, which plays key roles in regulating cancer cell proliferation, apoptosis, and cellular metabolism (17). FOXO1, in particular, has been reported to modulate several metabolic pathways, including glycolysis, gluconeogenesis, and glutamine metabolism in cancer and metabolic disorders (18). These functions suggest its potential involvement in regulating nitrogen metabolism and UC activity (19, 20, 21). Although FOXO1 and ASL dysregulation has been reported in various cancer types, including liver and colorectal cancers (22, 23), our analysis using the Kaplan–Meier Plotter database revealed that the inverse expression pattern—low FOXO1 and high ASL—is particularly prominent in metastatic breast cancer. This suggests that the regulatory relationship between FOXO1 and ASL may have specific relevance in the context of breast cancer progression. Given the role of FOXO1 as a metabolic regulator and transcription factor, we hypothesized that FOXO1 may directly regulate ASL expression in breast cancer. Previous studies on other FOXO family members (such as FOXO3a) regulating UC genes in different cancers further support the plausibility of this hypothesis (24).

In breast cancer, metastasis remains the major cause of mortality and is tightly associated with enhanced cellular migratory capacity. Epithelial–mesenchymal transition (EMT) represents a key mechanism driving this process. EMT is characterized by loss of epithelial traits and acquisition of mesenchymal features, enabling cancer cells to become more motile and invasive (25). During EMT, cells typically downregulate epithelial markers, such as E-cadherin, and upregulate mesenchymal markers, including N-cadherin and vimentin. These molecular changes facilitate cytoskeletal reorganization and extracellular matrix degradation, enabling cancer cells to detach from the primary tumor and migrate to distant organs (26). Monitoring EMT markers thus provides a valuable framework to assess how metabolic regulation by FOXO1–ASL signaling may influence metastatic behavior in breast cancer.

In this study, we investigated the role of FOXO1 in breast cancer and identified it as a transcriptional repressor of ASL. FOXO1 directly binds to the ASL promoter and suppresses its transcription, thereby limiting ASL expression and downstream ammonia metabolism. In addition, FOXO1 impedes breast cancer cell migration by modulating both ASL and arginine levels. Importantly, we show that supplementation with arginine can rescue the reduced migration caused by FOXO1-mediated suppression of ASL. These findings establish FOXO1 as a critical regulator in the UC and its potential role in controlling cancer cell migration.

## Results

### FOXO1 reduces the ammonia clearance ability of breast cancer cells and inhibits the expression of ASL

**Figure 1:**
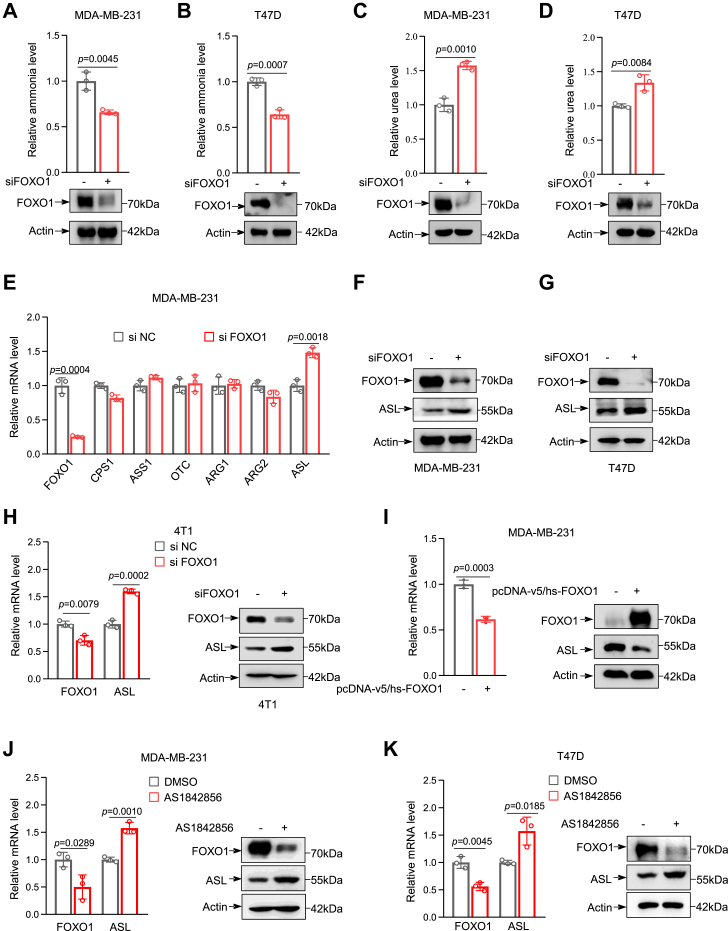
**FOXO1 reduces the ammonia clearance ability of breast cancer cells and inhibiting the expression of ASL.***A* and *B*, relative ammonia levels in MDA-MB-231 and T47D cells were measured 48 h after siRNA-mediated knockdown of FOXO1. *C* and *D*, relative urea levels in MDA-MB-231 and T47D cells were measured 48 h after siRNA-mediated knockdown of FOXO1. *E*, effect of FOXO1 silencing on the expression of urea cycle enzymes in MDA-MB-231 cells. *F* and *G*, Western blot analysis of FOXO1 and ASL protein levels in MDA-MB-231 and T47D cells 48 h after siRNA-mediated knockdown of FOXO1. Actin was used as a loading control. *H*, Western blot and quantitative RT–PCR (qRT–PCR) analyses of FOXO1 and ASL expression in 4T1 cells 48 h after siRNA-mediated knockdown of FOXO1. Actin was used as the loading control. *I*, effect of FOXO1 overexpression on ASL expression in MDA-MB-231 cells, as assessed by qRT–PCR and Western blot. *J*, the effect of the FOXO1 inhibitor AS1842856 on the expression of ASL, a urea cycle enzyme, in MDA-MB-231 cells was investigated by qRT–PCR and Western blot. *K*, the effects of the FOXO1 inhibitor AS1842856 on ASL expression in 4T1 cells were analyzed by qRT–PCR and Western blotting. Data are the mean ± SD. Each experiment was carried out at least three independent times. *p* Values were calculated by two-tailed unpaired Student’s *t* test. ∗*p* < 0.05, ∗∗*p* < 0.01, and ∗∗∗*p* < 0.001. ASL, argininosuccinate lyase; FOXO1, Forkhead box O1.

The UC is a key metabolic process within cells that detoxifies ammonia by converting it into urea. To explore the regulatory role of FOXO1 in UC metabolism in breast cancer, we first designed two FOXO1 siRNAs to evaluate their knockdown efficiency. The second siRNA, which showed the highest efficiency, was selected for subsequent experiments (Fig. S1*B*). After FOXO1 knockdown, we observed a remarkable decrease in ammonia production (Fig. 1, *A* and *B*), along with a significant increase in urea levels in MDA-MB-231 and T47D breast cancer cells (Fig. 1, *C* and *D*). In contrast, the overexpression of FOXO1 markedly increased ammonia production, accompanied by a decrease in urea levels in breast cancer cells (Fig. S1, *C–E*). To confirm the regulatory role of FOXO1 in UC metabolism, we assessed changes in key enzymes of the UC following FOXO1 knockdown in MDA-MB-231 cells. Notably, FOXO1 knockdown substantially enhanced the expression of the UC enzyme ASL, with no significant effect on other metabolic enzymes (Fig. 1*E*). Similar results were observed in T47D cells (Fig. S1*F*). The comparison of ASL expression levels between FOXO1 knockdown and wildtype cells indicated that the ASL expression level significantly increased after FOXO1 silencing (Fig. 1, *F*–*H*). Conversely, overexpression of FOXO1 led to a significant decrease in both mRNA and protein levels of ASL (Figs. 1*I* and S1*G*). Further validation of the inhibitory effect of FOXO1 on ASL expression was obtained by treating MDA-MB-231, T47D, and 4T1 cells with a FOXO1 antagonist, AS1842856, which also led to an increase in mRNA and protein expression levels of ASL (Figs. 1, *J*, *K* and S1*H*). This implies that FOXO1 is capable of repressing the expression of the UC metabolic enzyme ASL. The schematic diagram of the regulatory roles of the UC and FOXO1 in breast cancer is shown (Fig. S1*A*).

### *ASL* is a target gene for FOXO1

**Figure 2:**
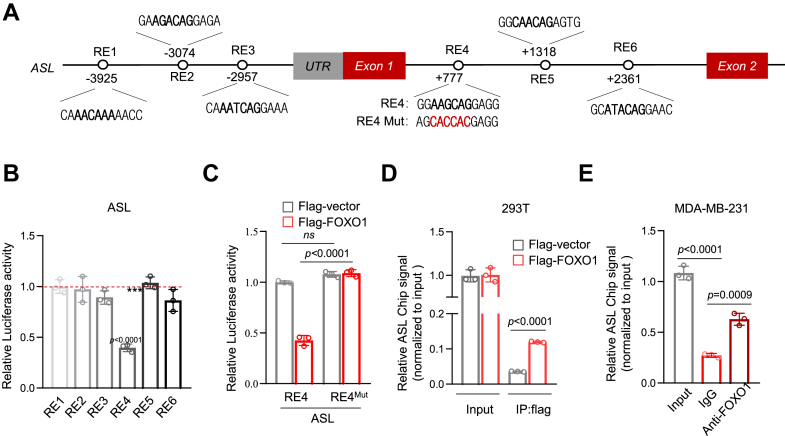
***ASL* is a target gene for FOXO1.***A*, schematic diagram of the human ASL genomic structure. The sequences of six potential FOXO1 response elements (RE1–RE6) and the corresponding mutated RE4 are shown. *B* and *C*, luciferase constructs containing RE1, RE2, RE3, RE4, RE5, and RE6 (*B*) or RE4 and mutant RE4 (*C*) were transfected into 293T cells together with FLAG-FOXO1 or vector control. Renilla vector pRL-CMV was used as a transfection internal control. The relative luciferase activity was normalized to the cotransfected Renilla activity and presented as the fold change relative to the vector control group (mean ± SD of two independent experiments), with the *Y*-axis representing normalized activity relative to the control. *D*, 293T cells transfected with control vector or FLAG-FOXO1 were subjected to ChIP assay using normal rabbit immunglobulin or anti-FLAG antibody. Bound DNA fragments were analyzed by PCR. *E*, MDA-MB-231 cells were subjected to ChIP assay using normal rabbit immunoglobulin or anti-FOXO1 antibody. Bound DNA was analyzed by PCR. Data are the mean ± SD. Each experiment was carried out at least three independent times. *p* Values were calculated by two-tailed unpaired Student’s *t* test or two-way ANOVA followed by Tukey’s multiple-comparison test. ∗*p* < 0.05, ∗∗*p* < 0.01, and ∗∗∗*p* < 0.001. ASL, argininosuccinate lyase; ChIP, chromatin immunoprecipitation; CMV, cytomegalovirus; FOXO1, Forkhead box O1.

FOXO1 is known to function as a transcription factor in various tumors (27, 28, 29). To evaluate the potential role of FOXO1 in modulating ASL, we examined the human FOXO1 gene sequence to determine the response elements (REs) of FOXO family proteins. These REs have a common sequence of 5′-GTAAACAG-3′. In addition, we detected three potential REs (RE1, RE2, and RE3) in the 5′ flanking region and three REs (RE4, RE5, and RE6) within the first intron (Fig. 2*A*). To explore the binding of FOXO1 to these REs, we cloned genomic fragments harboring each RE into luciferase reporter plasmids. The results showed that FOXO1 induced luciferase expression driven by RE4, rather than by RE1, RE2, RE3, RE5, or RE6 (Fig. 2*B*). Moreover, when RE4 was mutated, its luciferase expression was enhanced, with six conserved nucleotides being altered (Fig. 2*C*). Chromatin immunoprecipitation (ChIP) analysis indicated that both FLAG-FOXO1 (Fig. 2*D*) and endogenous FOXO1 (Fig. 2*E*) were associated with the RE4 region of FOXO1. Collectively, these results imply that FOXO1 binds to RE4 within ASL and represses ASL expression, suggesting that ASL is a target gene of FOXO1.

### FOXO1 suppresses ASL expression to impair ammonia clearance and regulate UC metabolites in breast cancer cells

**Figure 3:**
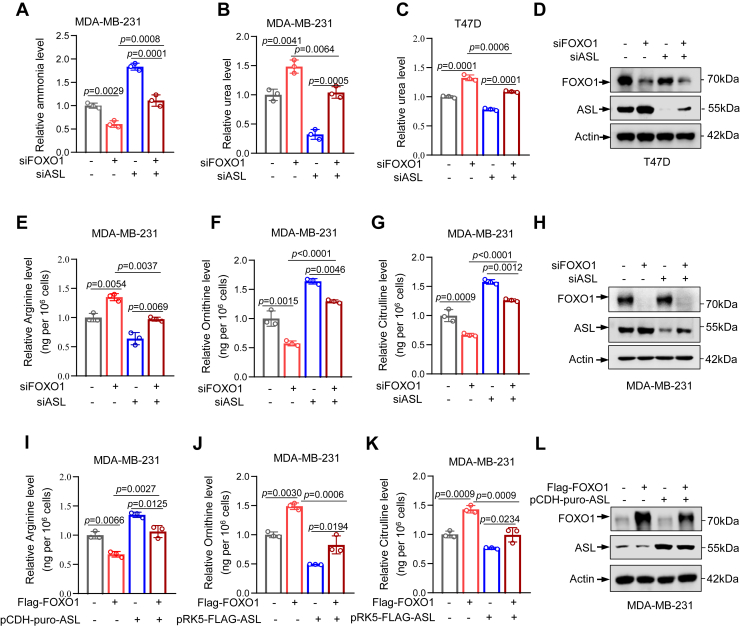
**FOXO1 suppresses ASL expression to impair ammonia clearance and regulate urea cycle metabolites in breast cancer cells.***A*–*C*, relative levels of ammonia and urea in MDA-MB-231 and T47D cells following siRNA-mediated knockdown of FOXO1 and ASL. *D*, Western blot analysis of protein levels in T47D cells after siRNA-mediated knockdown of FOXO1 and ASL. Actin was used as the loading control. *E*–*G*, relative abundances of urea cycle metabolites in MDA-MB-231 cells following siRNA-mediated knockdown of FOXO1 and ASL. *H*, Western blot analysis of protein levels in MDA-MB-231 cells after siRNA-mediated knockdown of FOXO1 and ASL. Actin was used as the loading control. *I*–*K*, relative abundances of urea cycle metabolites in MDA-MB-231 cells after overexpression of FOXO1 and ASL. *L*, Western blot analysis of FOXO1 and ASL protein levels in MDA-MB-231 cells after overexpression of FOXO1 and ASL. Actin was used as the loading control. Data are the mean ± SD. Each experiment was carried out at least three independent times. *p* Values were calculated by two-way ANOVA followed by Tukey’s multiple-comparison test. ∗*p* < 0.05, ∗∗*p* < 0.01, and ∗∗∗*p* < 0.001. ASL, argininosuccinate lyase; FOXO1, Forkhead box O1.

To further explore whether FOXO1 modulates the ammonia level and urea level in the UC through regulating ASL, we found that ASL knockdown in FOXO1-deficient cells restored intracellular ammonia levels and reversed the elevated urea production caused by FOXO1 silencing (Fig. 3, *A*–*D*). Next, we investigated whether FOXO1 affects metabolite levels downstream of the UC. We knocked down FOXO1 and ASL and quantified citrulline, arginine, and ornithine levels by LC–MS. Our data revealed that the knockdown of FOXO1 increased arginine levels while reducing ornithine and citrulline levels. In contrast, ASL knockdown decreased intracellular l-arginine levels but increased L-citrulline and L-ornithine. Interestingly, the concurrent knockdown of FOXO1 and ASL mitigated these changes, neutralizing the effects of FOXO1 loss alone (Fig. 3, *E*–*H*). However, overexpression of FOXO1 and ASL yielded opposite results (Fig. 3, *I*–*L*). These results indicate that FOXO1 suppresses UC metabolism by repressing ASL, leading to ammonia accumulation in breast cancer cells.

### FOXO1 suppresses the migration of breast cancer cells through modulating ASL

**Figure 4:**
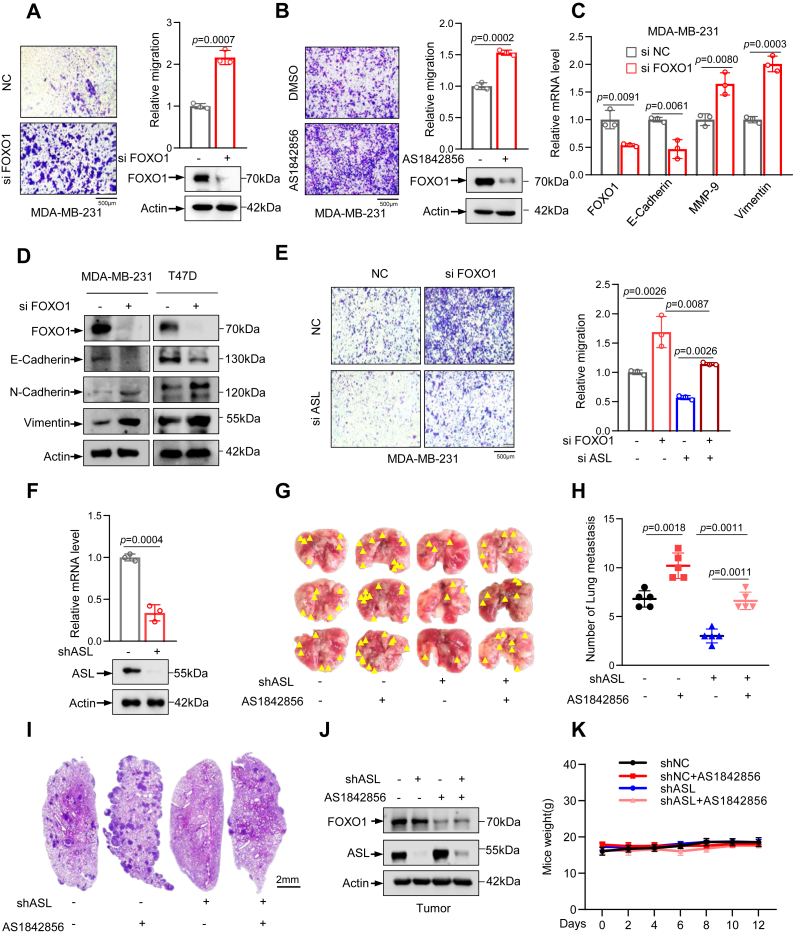
**FOXO1 suppresses the migration of breast cancer cells through modulating ASL.***A*, transwell migration assay was used to assess the migration of MDA-MB-231 cells after FOXO1 knockdown *via* siRNA. Representative images are shown on the *left*; quantification and Western blotting are shown on the *right*. The scale bar represents 500 μm (n = 3). *B*, transwell migration assay was used to assess the migration of MDA-MB-231 cells after treatment with the FOXO1 inhibitor AS1842856. Representative images are on the *left*; quantification and Western blotting are on the *right*. The scale bar represents 500 μm (n = 3). *C*, FOXO1 was knocked down in MDA-MB-231 cells, and relative expression levels of EMT-associated genes were quantified by quantitative qRT–PCR. *D*, Western blotting analysis of FOXO1 and EMT-associated proteins following FOXO1 knockdown. *E*, transwell migration assay was performed to assess the migration of MDA-MB-231 cells with simultaneous knockdown of FOXO1 and ASL. Representative images are shown on the *left*, and quantification is presented on the *right*. The scale bar represents 500 μm (n = 3). *F*, an ASL-knockout 4T1 cell line was constructed, and ASL expression was analyzed by quantitative RT–PCR and Western blotting. *G* and *H*, ASL-deficient 4T1 cells were injected into BALB/c mice *via* the tail vein, followed by daily intraperitoneal administration of the FOXO1 inhibitor AS1842856 for 12 consecutive days. Mice were sacrificed on day 12, and lung metastases were evaluated and quantified (n = 5 per group). *Yellow arrowheads* in (*G*) indicate visible metastatic nodules on the lung surface. *I*, representative H&E staining of lung sections from the experimental groups shown in (*G*). hematoxylin stains cell nuclei *dark purple*, highlighting the dense nuclear morphology of tumor cells. Eosin stains the cytoplasm and extracellular matrix *pink*. The presence of *dark purple dots* (tumor cell nuclei) within eosin-stained lung parenchyma indicates metastatic lesions. The scale bar represents 2 mm. *J*, protein levels of FOXO1 and ASL in lung metastatic tissues were analyzed by Western blotting. *K*, body weight of the mice was recorded. Data are the mean ± SD. Each experiment was carried out at least three independent times. *p* Values were calculated by two-tailed unpaired Student’s *t* test or two-way ANOVA followed by Tukey’s multiple-comparison test. ∗*p <* 0.05, ∗∗*p* < 0.01, and ∗∗∗*p* < 0.001. ASL, argininosuccinate lyase; EMT, epithelial–mesenchymal transition; FOXO1, Forkhead box O1.

To investigate the effects of FOXO1 on tumor cell migration, we silenced FOXO1 in MDA-MB-231 and 4T1 cells using siRNA and conducted transwell assays. FOXO1 knockdown significantly enhanced cell migration (Figs. 4*A* and S2*A*), whereas its overexpression markedly reduced the migratory potential of breast cancer cells (Fig. S2*B*). Further validation using the FOXO1 antagonist AS1842856 in MDA-MB-231, T47D, and 4T1 cells confirmed that FOXO1 inhibition accelerated cell migration (Figs. 4*B* and S2, *C*, *D*). Subsequently, we examined the effect of FOXO1 knockdown on the EMT process in breast cancer cells. Our research findings indicate that FOXO1 knockdown inhibited the expression of epithelial markers while promoting the expression of mesenchymal markers. By contrast, FOXO1 overexpression was found to suppress EMT, as evidenced by the reduced expression of E-cadherin and increased vimentin, N-cadherin, and MMP-9 expression (Figs. 4, *C*, *D* and S2, *E*, *F*). These findings demonstrate that FOXO1 inhibits breast cancer cell migration and mitigates the EMT process, highlighting its critical role in suppressing tumor progression.

Next, we examined the role of FOXO1-mediated ASL suppression in breast cancer cell migration. We first observed that ASL knockdown significantly reduced migratory capacity of breast cancer cells, whereas ASL overexpression remarkably enhanced it (Fig. S2, *G* and *H*). Similarly, ASL knockdown promotes the expression of epithelial-related markers and inhibits the expression of mesenchymal markers, whereas ASL overexpression yields the opposite result (Fig. S2, *I* and *J*), contrasting with the EMT-promoting effect of FOXO1 knockdown. Notably, the migration ability of tumor cells is attenuated after further knockdown of ASL in MDA-MB-231 cancer cells with FOXO1 knockdown (Fig. 4*E*). The identical results were obtained in T47D cells (Fig. S2*K*). Conversely, in MDA-MB-231, T47D, and 4T1 cells, after overexpression of FOXO1 and further overexpression of ASL, the migration ability of tumor cells was promoted (Fig. S2, *L*–*N*). These results demonstrate that FOXO1 inhibits the migration of breast cancer cells by downregulating ASL and mitigating EMT progression.

To further assess the antimetastatic potency of FOXO1, we constructed a 4T1 ASL knockout cell line (Fig. 4*F*) and established a breast cancer lung metastasis model in female BALB/c mice *via* tail vein. Mice were intraperitoneally injected with AS1842856 or a vehicle control once daily. The results show that treatment with the FOXO1 inhibitor significantly promoted breast cancer lung metastasis, as evidenced by an increased number of metastatic nodules on the lung surface. In contrast, ASL deletion markedly suppressed lung metastasis, reducing the number of metastatic nodules. Importantly, further knockout of ASL in FOXO1-knockdown cells reduced the enhanced lung metastasis induced by FOXO1 inhibition (Fig. 4, *G*–*I*), highlighting that ASL functions as a downstream effector of FOXO1 that mediates its suppressive effects on metastasis through the regulation of arginine metabolism. In addition, the expressions of FOXO1 and ASL in the lung tissues of mice were also detected (Fig. 4*J*). After 12 days of treatment, no significant difference in body weight was observed among the four groups (Fig. 4*K*).

### ASL promotes breast cancer cell migration by regulating arginine

Previous studies have shown that arginine deprivation has the potential to attenuate the metastatic ability of pancreatic cancer cells (30). Similar to previous studies, our results demonstrated that arginine supplementation remarkably enhanced the aggregation and migratory capacities of breast cancer cells (Fig. S3, *A–C*). Subsequently, we investigated the effect of arginine supplementation on the EMT process of breast cancer cells. Our research results show that supplementation of arginine can inhibit the expression of epithelial markers and promote the expression of interstitial markers simultaneously (Fig. S3, *D–G*), indicating its role in facilitating the EMT. In contrast, ornithine and citrulline supplementation did not significantly affect the migratory behavior of breast cancer cells (Fig. S3, *H–K*).

**Figure 5:**
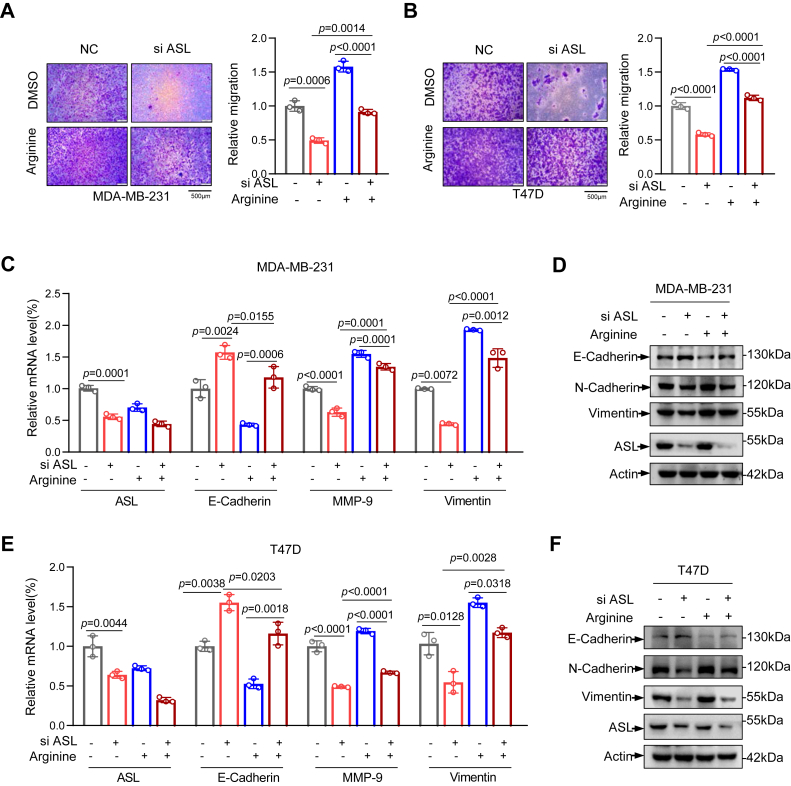
**ASL promotes breast cancer cell migration by regulating arginine.***A* and *B*, transwell migration assays were performed to assess migration of MDA-MB-231 and T47D cells after ASL knockdown by siRNA, under conditions with or without 10 mM arginine treatment. Representative images are on the *left*; quantification is shown on the *right*. The scale bar represents 500 μm (n = 3). *C* and *E*, MDA-MB-231 and T47D cells with ASL knockdown were treated with or without 10 mM arginine for 48 h. EMT-related gene expression was quantified by quantitative RT–PCR. *D* and *F*, ASL-knockdown MDA-MB-231 and T47D cells treated with or without 10 mM arginine for 48 h were analyzed by Western blotting for ASL and EMT-associated proteins. Data are the mean ± SD. Each experiment was carried out at least three independent times. *p* Values were calculated by two-way ANOVA followed by Tukey’s multiple-comparison test. ∗*p <* 0.05, ∗∗*p* < 0.01, and ∗∗∗*p <* 0.001. ASL, argininosuccinate lyase; EMT, epithelial–mesenchymal transition.

In light of the fact that arginine is one of the downstream metabolites in the UC regulated by ASL and our results indicated that arginine can promote the migration of breast cancer cells, we postulate that ASL may affect the migration of breast cancer cells by regulating arginine levels. To validate this hypothesis, we conducted transwell assays and found that knockdown of ASL restricts the migration of breast cancer cells, whereas arginine supplementation accelerates the migration of the cells (Fig. 5, *A* and *B*). Further validation through quantitative PCR (qPCR) and Western blot analysis revealed that ASL knockdown increased epithelial marker expression and suppressed interstitial marker expression, whereas arginine supplementation had the opposite effect. Notably, the combination of ASL knockdown and arginine supplementation counteracted the reduced migration seen with ASL knockdown alone (Fig. 5, *C*–*F*). These results suggest that ASL may promote breast cancer cell migration by modulating arginine metabolism, although we cannot exclude alternative mechanisms, such as altered UC flux upstream of ASL through arginase activity.

### FOXO1 suppresses the migration of breast cancer cells through modulating ASL and arginine

**Figure 6:**
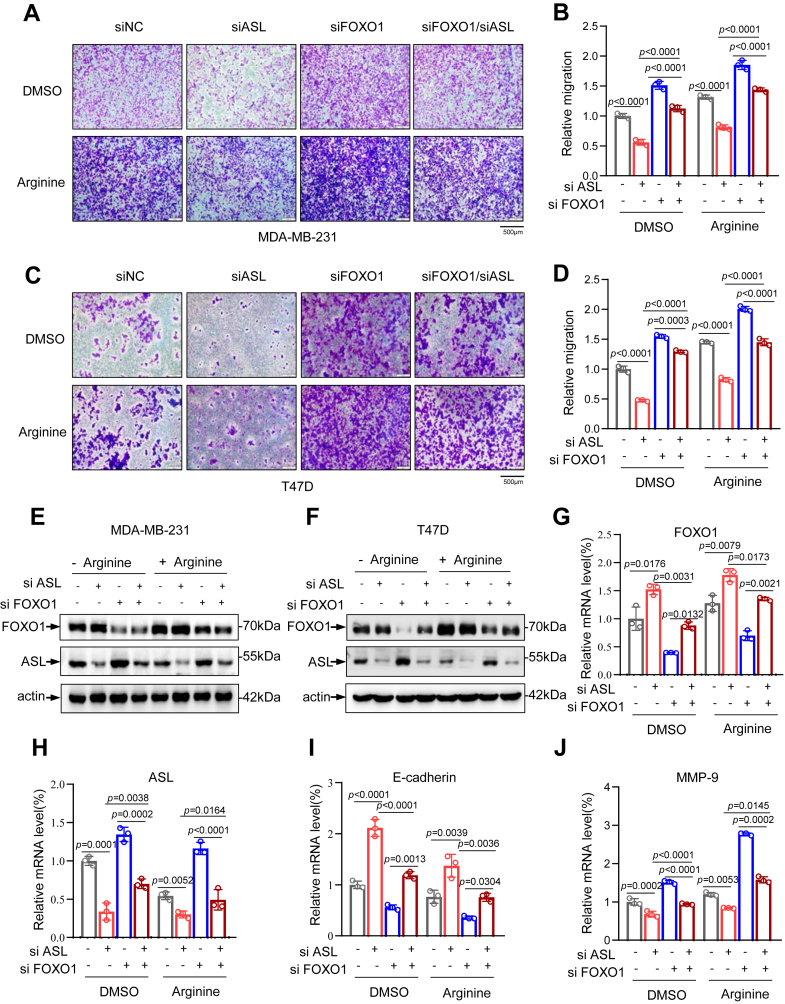
**FOXO1 suppresses the migration of breast cancer cells through modulating ASL and arginine.***A*–*D*, migration of FOXO1- and ASL-silenced MDA-MB-231 and T47D cells with or without arginine supplementation was assessed using transwell assays. Representative images are shown on the *left*; quantitative data are presented on the *right*. The scale bar represents 500 μm (n = 3). *E* and *F*, Western blot analysis of protein levels in MDA-MB-231 and T47D cells following siRNA-mediated knockdown of FOXO1 and ASL, under conditions with or without 10 mM arginine treatment. Actin was used as a loading control. *G*–*J*, FOXO1 and ASL were knocked down in MDA-MB-231 cells, with or without 10 mM arginine treatment. The expression of FOXO1, ASL, and EMT-associated genes was quantified by quantitative RT–PCR. Data are the mean ± SD. Each experiment was carried out at least three independent times. *p* Values were calculated by two-way ANOVA followed by Tukey’s multiple-comparison test. ∗*p* < 0.05, ∗∗*p* < 0.01, and ∗∗∗*p* < 0.001. ASL, argininosuccinate lyase; EMT, epithelial–mesenchymal transition; FOXO1, Forkhead box O1.

Subsequently, we evaluated how FOXO1-mediated regulation of ASL and its downstream metabolite arginine affects breast cancer cell migration using transwell. As expected, FOXO1 knockdown enhanced the migratory capacity of breast cancer cells. In contrast, silencing ASL reduced cell migration. Moreover, simultaneous knockdown of ASL in FOXO1-deficient cells further attenuated their migratory ability. Notably, arginine supplementation restored the migration of breast cancer cells (Fig. 6, *A*–*F*). Consistently, qPCR analysis showed that FOXO1 downregulation reduced epithelial marker expression and increased mesenchymal marker expression. Conversely, ASL knockdown produced the opposite pattern, with elevated epithelial markers and suppressed mesenchymal markers. Furthermore, arginine supplementation markedly upregulated epithelial markers while generally downregulating mesenchymal markers (Fig. 6, *G*–*J*). Together, these findings suggest that FOXO1 inhibits breast cancer cell migration through regulation of ASL and its downstream metabolite, arginine.

### Low expression of FOXO1 correlates with unfavorable prognosis in breast cancer patients

**Figure 7:**
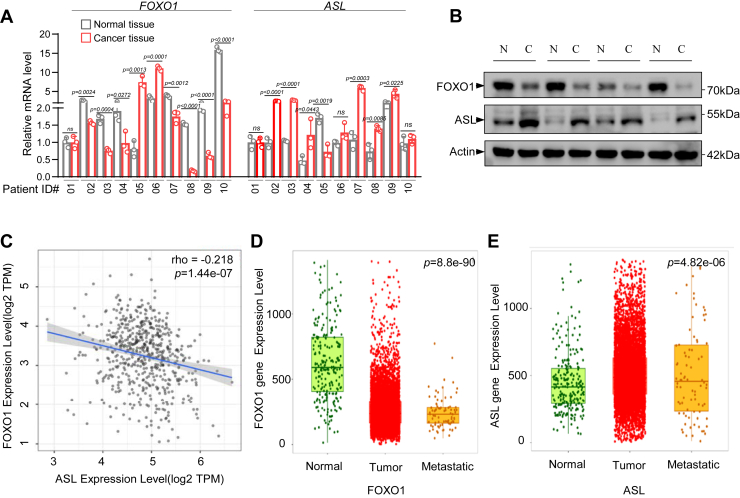
**Low expression of FOXO1 correlates with unfavorable prognosis in breast cancer patients.***A*, mRNA expression levels of FOXO1 and ASL in paired human breast cancer tissues and adjacent normal tissues (n = 10) were analyzed by RT–quantitative PCR. Data are presented as mean ± SD. Statistical analysis: two-tailed paired Student’s *t* test. *B*, representative Western blot images showing protein expression of FOXO1 and ASL in paired cancer (C) and normal (N) tissues from breast cancer patients (n = 4). Actin was used as a loading control. *C*, correlation analysis between FOXO1 and ASL expression levels in breast cancer samples from the TIMER2.0 dataset (n = 1100). Expression values are shown as log_2_ transcript per million (TPM). *Statistical analysis*: Spearman’s rank correlation coefficient (ρ); *p* value indicated. *D* and *E*, transcriptome data showing FOXO1 (*D*) and ASL (*E*) gene expression levels in normal breast tissue (n = 242), primary breast cancer (n = 7569), and metastatic breast cancer tissues (n = 82), retrieved from the Kaplan–Meier Plotter database. *Statistical analysis*: Kruskal–Wallis test followed by Dunn’s *post hoc* test. Data are the mean ± SD. ∗*p* < 0.05, ∗∗*p* < 0.01, and ∗∗∗*p* < 0.001. Each experiment was performed in at least three independent replicates unless otherwise stated. ASL, argininosuccinate lyase; FOXO1, Forkhead box O1.

To explore the expression of FOXO1 and ASL in human cancers and their potential correlation, we analyzed 10 pairs of breast cancer tissues and matched normal tissues. Intriguingly, we found that FOXO1 mRNA expression was low in seven of the 10 breast cancer samples, whereas ASL expression was elevated (Fig. 7*A*). Subsequently, the protein expression levels of FOXO1 and ASL in four pairs of breast cancer tissues and adjacent tissues were compared. The results demonstrated that the expression level of FOXO1 in breast cancer tissues was low, whereas the expression level of ASL was high (Fig. 7*B*). Analysis of the TIME2.0 tumor database and the Kaplan–Meier Plotter revealed a negative correlation between FOXO1 and ASL expression (Fig. 7*C*), and the expression level of FOXO1 in breast cancer tissue and cancer metastasis tissues is low (Fig. 7*D*). In contrast, the expression of ASL was markedly upregulated in breast cancer tissues and cancer metastasis tissues (Fig. 7*E*). These findings suggest that in breast cancer tissues, FOXO1 expression is downregulated, whereas ASL expression is upregulated. Moreover, an inverse correlation is presented between the two.

## Discussion

FOXO1 is a transcription factor known to regulate various biological processes, including proliferation, apoptosis, cell cycle, and autophagy (27, 31). In our study, FOXO1 functions as a tumor suppressor in breast cancer, as evidenced by its lower expression in poor-prognosis patients and its inhibitory effect on breast cell migration both *in vitro* and *in vivo*.

Tumor cells undergo metabolic reprogramming to optimize nitrogen and carbon utilization for growth and migration. The UC, a key pathway for nitrogen disposal, is often altered in cancer (32). ASL catalyzes the conversion of argininosuccinate into arginine and fumarate, playing a key role in arginine biosynthesis (13). Elevated ASL expression correlates with poor prognosis in several cancers, and its inhibition reduces cancer cell proliferation. ASL-derived arginine serves as a substrate for nitric oxide (NO) synthase, facilitating NO and citrulline production. Subsequently, NO serves as a powerful signaling molecule that modulates cancer cell functions, such as proliferation, angiogenesis, and EMT, in a dose-dependent manner. Arginine depletion has arisen as a novel therapeutic approach, efficiently restraining cancer cell migration and invasion. The knockdown of ASL suppresses the EMT process in breast cancer cells. Conversely, the downregulation of FOXO1 augments ASL expression and drives EMT. In our study, E-cadherin, N-cadherin, and vimentin were assessed as key EMT markers to determine the effect of FOXO1–ASL signaling on cellular phenotype. Overexpression of FOXO1 increased E-cadherin while decreasing N-cadherin and vimentin levels, indicative of EMT suppression and reduced metastatic potential. In contrast, FOXO1 knockdown upregulated ASL, thereby enhancing arginine metabolism and promoting EMT. These results identify ASL as a critical downstream effector through which FOXO1 regulates EMT, likely *via* arginine-dependent pathways. This regulatory effect may involve FOXO1’s control over ASL-related metabolites, such as NO and arginine, influencing their biosynthesis and, in turn, EMT progression. Together with previous findings that ASS1 silencing supports pyrimidine synthesis and proliferation through aspartate redirection (9), our data further emphasize the importance of UC enzymes in reprogramming tumor nitrogen metabolism and regulating malignant behaviors. FOXO1 governs the migration of breast cancer cells through the transcriptional repression of ASL expression, thereby clarifying its essential role in the regulation of tumor cell ammonia metabolism.

The metastatic colonization of breast cancer cells in distant organs such as the liver and lungs requires substantial metabolic reprogramming to adapt to distinct microenvironments (7, 33). These sites show markedly different UC activity, which may influence how FOXO1-mediated repression of ASL affects tumor progression. Although the liver is intrinsically rich in UC enzymes (34), recent studies indicate that in the context of cancer, hepatic UC activity can be significantly diminished. For example, in breast cancer–bearing mice, the liver exhibits reduced UC flux and increased uracil production, suggesting that systemic tumor burden can reprogram liver metabolism even in the absence of direct metastasis (35). Notably, FOXO1 expression is reduced in some liver malignancies (36, 37), possibly as an adaptive mechanism to maintain ASL activity.

Unlike the liver, the lung and bone exhibit low baseline expression of UC enzymes and limited capacity for arginine recycling, making tumor cells more reliant on endogenous arginine synthesis (32, 38, 39). To adapt, metastatic cells in these niches repurpose UC intermediates for biosynthetic demands. Recent metabolic flux analyses show that lung-homing breast cancer sublines (LM) display a highly glycolytic phenotype with attenuated mitochondrial respiration, increased demand for nucleotide synthesis, and enriched nonoxidative pentose phosphate pathway activity (40). Specifically, elevated ASL expression in breast tumors likely enhances arginine production (41), supporting NO (42) and polyamine synthesis (8), or redirecting metabolism toward pyrimidine synthesis (43). In bone metastases, ASL upregulation may supply arginine for NO production, which modulates osteoclast activity, immunity, and vascularization in bone lesions (44). FOXO1-mediated repression of ASL could reduce arginine-driven NO synthesis, thereby impairing osteoclast–tumor interactions that promote osteolysis. Consequently, FOXO1-driven ASL suppression may sensitize lung metastases to arginine limitation, restricting colonization and outgrowth.

In summary, our study reveals that FOXO1 inhibits breast cancer cell migration by repressing ASL expression and disrupting ammonia and arginine metabolism. This regulatory axis offers insight into the metabolic control of metastasis and suggests FOXO1 as a potential target for therapeutic intervention in breast cancer.

## Experimental procedures

### Human breast cancer and paracancerous tissue

The study was approved by the Ethical Committee of General Hospital of Ningxia Medical University (protocol number: KYLL-2024-0486). All relevant ethical regulations were followed in this study. Human tissues were obtained from patients at the General Hospital of Ningxia Medical University. All patients received written informed consent. Breast and paracancer tissues were collected from 10 patients. This research adheres to the Declaration of Helsinki.

### Mice

Female BALB/c nude mice (4–5 weeks old) were purchased from Skobes Creatures Co, Ltd. For metastasis studies, 1 × 10^6^ 4T1 and 4T1 ASL knockout cells were injected intravenously into the tail veins of 5- to 6-week-old female nude mice (n = 5 per group). The animals were maintained in a specific pathogen-free facility under a 12-h dark–light cycle at a temperature of 24 ± 1 °C with free access to a standard rodent diet and water. Mice were treated with either 15 mg/kg AS1842856 (Adooq Bioscience; A15871) or vehicle (6% 2-hydroxypropyl-β-cyclodextrin and 5% dimethyl sulfoxide in saline) once per day for 12 days. Animal weights were measured every other day. After 12 days, mice were euthanized by cervical dislocation, and lungs were harvested, fixed in paraformaldehyde, and processed for H&E staining to assess metastasis. All animal analyses were performed in a blinded manner to minimize bias.

### Cell culture

Human breast cancer cell lines T47D and MDA-MB-231 were kindly provided by Prof Peng Jiang (Tsinghua University). 4T1 cells were purchased from ServiceBio Company. The MDA-MB-231 cells were cultured in Dulbecco's modified Eagle's medium (Gibco) supplemented with 10% fetal bovine serum (Gemini), 100 U/ml penicillin, and 100 μg/ml streptomycin at 37 °C in an atmosphere of 5% CO_2_. MDA-MB-231 cells were cultured in Dulbecco's modified Eagle's medium (Gibco), 4T1 cells were cultured in RPMI1640 (Gibco), with both media supplemented with 10% fetal bovine serum (Gemini) and 1% penicillin–streptomycin (Thermo Fisher). All cells were cultured at 37 °C with 5% CO_2_. Silencing of ASL in 4T1 cells was performed by stably transfecting plasmids containing relative shRNA. The shRNA ASL murine plasmid·was purchased from·Miao Ling Biotechnology.

### FOXO1 or ASL knockdown

To knock down FOXO1 or ASL, the siRNA used in this study was synthesized by Gene Pharma. The siRNA transfection is performed according to the manufacturer's instructions. For 6-well plates, a mixture of 3 μl RNAiMAX (Thermo Scientific) and 250 μl Opti-MEM was prepared and incubated for 5 min. siRNA (2 μl; 20 nm) was mixed with 250 μl Opti-MEM for 5 min. The mixture is then mixed and incubated at room temperature for 15 min. The resulting mixture was uniformly added to the cells with a mix of 30%. The culture medium is changed 24 h after transfection, and various treatments can be performed if necessary. Subsequent experiments were performed 48 h after transfection.

siNC: 5′-UUCUCCGAACGUGUCACGUTT-3′, siFOXO1 #1: 5′-GGAGGUAUGAGUCAGUAUATT-3′, siFOXO1 #2: 5′-GCCCUGGCUCUCACAGCAATT-3′, siASL#1: 5′-CCUUCAAACUGAACUCCAATT-3′ siASL#2: 5′-AGGAGGCUGCUGUGUGUUUTT-3′.

### Transwell assay

The migratory behavior of breast cancer cells was assessed through the ttranswell assay. MDA-MB-231, T47D, and 4T1 cells (5 × 10^4^ cells/well) resuspended in serum-free medium were seeded into the upper chamber of 24-well transwell plates (Corning Costar). The cells were plated in 300 μl serum-free medium in the upper chambers, and 700 μl medium supplemented with 20% fetal bovine serum was placed into the lower chambers. After 24 h, the cells on the upper chamber were removed, and the cells on the lower chamber were fixed by 4% polyformaldehyde and stained with crystal violet. The images were photographed, and the cell number was counted.

### Western blot

Cell cultures and tissues were lysed using radioimmunoprecipitation assay buffer, and the protein concentration was quantified using a bicinchoninic acid kit from Sangon. Extracted proteins were separated on 10% SDS-PAGE gels and transferred to a nitrocellulose membrane (Millipore). The membranes underwent blocking using 5% skimmed milk followed by an overnight incubation at 4 °C with primary antibodies. Subsequently, secondary antibodies were applied at room temperature for 1 h, after which chemiluminescence detection was performed. Antibodies against FOXO1 (CST; 2880), ASL (Proteintech; 16645-1-AP), E-cadherin (CST; 14472), N-cadherin (CST; 13116), vimentin (CST; 5741), and β-actin (CST; 3700) at 4 °C overnight. All the secondary antibodies were from EASYBIO.

### Quantitative real-time PCR

Total RNA was isolated from the cells using an RNA extraction kit (DAKEW). Subsequently, reverse transcription was conducted using the PrimeScript RT reagent Kit with gDNA Eraser kit (Takara), following the manufacturer's instructions. Quantification of targeted genes was determined with TransStart Top Green qPCR SuperMix (TransGen Biotech). mRNA level is normalized to β-actin. Primer sequences are listed as follows:

FOXO1: 5′-CCAGCCCAAACTACCAAAAATA-3′ and 5′-GAGGAGAGTCAGAAGTCAGCAAC-3′; ASL: 5′-CCTTTGGGAGGTGGATGT-3′ and 5′-CTCATTGGCTGTGTGGATG-3′; β-actin: 5′-GTCTTCCCCTCCATCGTG-3′ and 5′-AGGGTGAGGATGCCTCTCTT-3′.

### ChIP and luciferase assay

We utilized JASPAR1 (http://jaspar.genereg.net) to pinpoint potential FOXO1 REs within ASL genes. To conduct ChIP assays, cellular entities were subjected to crosslinking employing 1% formaldehyde for a duration of 15 min at room temperature, with cessation of crosslinking achieved through the addition of 125 nM glycine to attain a final concentration. Subsequently, cellular lysates underwent sonication to produce DNA fragments of an average size below 1000 bp, which were then subjected to immunoprecipitation utilizing specified antibodies. The captured DNA fragments were later released and amplified through PCR techniques. For the reporter assay, the DNA sequences corresponding to FOXO1 REs within the *ASL* gene, namely RE1, RE2, RE3, RE4, RE5, RE6, the wildtype, and the mutant FOXO1 binding region (RE4), were inserted into the pGL3-basic vector (Promega). Luciferase activity was evaluated utilizing a dual-luciferase assay system (Promega), with transfection efficiency standardized to Renilla luciferase activity.

### Metabolite extraction and mass spectrometry–based metabolomics analysis

MDA-MB-231 cells (4 × 10^6^ cells) were transfected with siRNA targeting FOXO1–ASL knockdown, collected in cold PBS using cell scrapers, and then centrifuged at 12,000 rpm for 10 min in a high-speed refrigerated centrifuge. The supernatant was removed, and the sediment was quickly frozen using liquid nitrogen. Finally, the samples were stored for subsequent metabolomic analyses. The cellular residue was subjected to a solution comprising precooled methanol, acetonitrile, and water (in a volume ratio of 2:2:1, 1 ml) and subsequently ultrasonicated in an ice bath for 1 h to facilitate the extraction of metabolites. After incubating the mixture at −20 °C for 1 h, it was centrifuged in a high-speed refrigerated centrifuge at 14,000 rpm for 20 min. Subsequently, the resulting samples were transferred to a sample bottle for LC–MS analysis. The LC–MS portion of the platform was based on the Shimadzu Nexera X2 LC-30AD system equipped with an ACQUITY UPLC BEH Amide column (1.7 μm, 2.1 mm × 100 mm; Waters) and a triple quadrupole mass spectrometer (5500 QTRAP; AB SCIEX). The metabolites were identified using electrospray ionization in both negative and positive modes. Approximately 2 μl of the samples were sequentially injected using an LC autosampler. The ACQUITY UPLC BEH Amide column (1.7 μm, 2.1 mm × 100 mm; Waters) was maintained at 45 °C while running at a flow rate of 300 μl/min. A gradient method was employed to separate and purify the samples. The raw multiple reaction monitoring data were processed and analyzed using the MultiQuant software (AB SCIEX). The raw data underwent normalization employing a statistically significant threshold determined by fold change (FC) and a two-tailed Student’s *t* test (*p* value) to differentiate metabolites. FC was calculated as the logarithm of the average mass response (area) ratio between two distinct classes. The *p* value was calculated using one-way ANOVA for multiple-group analysis. Metabolites were deemed statistically significant when exhibiting an FC exceeding 1.5 and a *p* value below 0.05. The identified differential metabolites underwent cluster analysis employing the R package for further examination.

### Data analysis on public datasets

The correlation between FOXO1 and ASL mRNA expression in breast cancer samples was analyzed using the TIMER2.0 online platform (http://timer.cistrome.org/). The correlation plot (n = 1100) was generated using default settings, with results presented as log_2_ transcript per million values and evaluated using Spearman’s rank correlation coefficient. FOXO1 and ASL mRNA expression levels in normal breast tissue, primary breast cancer, and metastatic breast cancer tissues were visualized using the Kaplan–Meier Plotter online database (https://kmplot.com/analysis/). Expression levels were compared using the Kruskal–Wallis test with Dunn’s *post hoc* test, as performed by the platform.

### Measurements of ammonia and urea

The ammonia quantitative kit (abcam) was used for cell ammonia analysis. The urea quantification Kit (abcam) was used and followed the manufacturer's instructions.

### Statistical analysis

All experiments were repeated at least three times. Column graphs were generated using GraphPad Prism, v8.0.1 (GraphPad Software, Inc). Statistical significance was calculated using the unpaired two-tailed *t* test or two-way ANOVA, followed by Tukey's multiple comparison test (not significant). The difference was statistically significant, *p* < 0.05.

## Data availability

The metabolomics data generated and analyzed during this study have been deposited in [“Figshare”]. As the Kaplan–Meier Plotter and TIMER2.0 analyses were performed using online platforms, the original source data are available at Kaplan–Meier Plotter and TIMER2.0.

## Supporting information

This article contains supporting information.

## Conflict of interest

The authors declare that they have no conflicts of interest with the contents of this article.

## References

[1] Stine Z.E., Schug Z.T., Salvino J.M., Dang C.V. (2022). Targeting cancer metabolism in the era of precision oncology. Nat. Rev. Drug Discov..

[2] Vander Heiden M.G., Cantley L.C., Thompson C.B. (2009). Understanding the Warburg effect: the metabolic requirements of cell proliferation. Science.

[3] Hanahan D., Weinberg R.A. (2011). Hallmarks of cancer: the next generation. Cell.

[4] Spinelli J.B., Yoon H., Ringel A.E., Jeanfavre S., Clish C.B., Haigis M.C. (2017). Metabolic recycling of ammonia via glutamate dehydrogenase supports breast cancer biomass. Science (New York, NY).

[5] Wilkinson D.J., Smeeton N.J., Watt P.W. (2010). Ammonia metabolism, the brain and fatigue; revisiting the link. Prog. Neurobiol..

[6] Cheng C., Geng F., Li Z., Zhong Y., Wang H., Cheng X. (2022). Ammonia stimulates SCAP/Insig dissociation and SREBP-1 activation to promote lipogenesis and tumour growth. Nat. Metab..

[7] Keshet R., Szlosarek P., Carracedo A., Erez A. (2018). Rewiring urea cycle metabolism in cancer to support anabolism. Nat. Rev. Cancer.

[8] Li L., Mao Y., Zhao L., Li L., Wu J., Zhao M. (2019). p53 regulation of ammonia metabolism through urea cycle controls polyamine biosynthesis. Nature.

[9] Rabinovich S., Adler L., Yizhak K., Sarver A., Silberman A., Agron S. (2015). Diversion of aspartate in ASS1-deficient tumours fosters de novo pyrimidine synthesis. Nature.

[10] Arruabarrena-Aristorena A., Zabala-Letona A., Carracedo A. (2018). Oil for the cancer engine: the cross-talk between oncogenic signaling and polyamine metabolism. Sci. Adv..

[11] Gerner E.W., Meyskens F.L. (2004). Polyamines and cancer: old molecules, new understanding. Nat. Rev. Cancer.

[12] Huang H.L., Hsu H.P., Shieh S.C., Chang Y.S., Chen W.C., Cho C.Y. (2013). Attenuation of argininosuccinate lyase inhibits cancer growth via cyclin A2 and nitric oxide. Mol. Cancer Ther..

[13] Huang H.L., Chen W.C., Hsu H.P., Cho C.Y., Hung Y.H., Wang C.Y. (2017). Silencing of argininosuccinate lyase inhibits colorectal cancer formation. Oncol. Rep..

[14] Al-Koussa H., Al-Haddad M., Abi-Habib R., El-Sibai M. (2019). Human recombinant arginase I [HuArgI (Co)-PEG5000]-Induced arginine depletion inhibits colorectal cancer cell migration and invasion. Int. J. Mol. Sci..

[15] Al-Koussa H., El Mais N., Maalouf H., Abi-Habib R., El-Sibai M. (2020). Arginine deprivation: a potential therapeutic for cancer cell metastasis? A review. Cancer Cell Int..

[16] Yuan Y., Li H., Pu W., Chen L., Guo D., Jiang H. (2022). Cancer metabolism and tumor microenvironment: fostering each other?. Sci. China Life Sci..

[17] Gui T., Burgering B.M.T. (2022). FOXOs: masters of the equilibrium. FEBS J..

[18] Dong X.C., Copps K.D., Guo S., Li Y., Kollipara R., DePinho R.A. (2008). Inactivation of hepatic Foxo1 by insulin signaling is required for adaptive nutrient homeostasis and endocrine growth regulation. Cell Metab..

[19] Link W., Fernandez-Marcos P.J. (2017). FOXO transcription factors at the interface of metabolism and cancer. Int. J. Cancer.

[20] Nakamura M.T., Yudell B.E., Loor J.J. (2014). Regulation of energy metabolism by long-chain fatty acids. Prog. Lipid Res..

[21] Jung S.M., Hung C.M., Hildebrand S.R., Sanchez-Gurmaches J., Martinez-Pastor B., Gengatharan J.M. (2019). Non-canonical mTORC2 signaling regulates brown adipocyte lipid catabolism through SIRT6-FoxO1. Mol. Cell.

[22] Zhang Y., Wang H., Wang Y., Ma B. (2025). FOXO1 mediates miR-99a-5p/E2F7 to restrain breast cancer cell proliferation and induce apoptosis. BMC Cancer.

[23] Huang H.L., Chen W.C., Hsu H.P., Cho C.Y., Hung Y.H., Wang C.Y. (2015). Argininosuccinate lyase is a potential therapeutic target in breast cancer. Oncol. Rep..

[24] Sun W., Kou H., Fang Y., Xu F., Xu Z., Wang X. (2024). FOXO3a-regulated arginine metabolic plasticity adaptively promotes esophageal cancer proliferation and metastasis. Oncogene.

[25] Dongre A., Weinberg R.A. (2019). New insights into the mechanisms of epithelial-mesenchymal transition and implications for cancer. Nat. Rev. Mol. Cel. Biol..

[26] Fontana R., Mestre-Farrera A., Yang J. (2024). Update on epithelial-mesenchymal plasticity in cancer progression. Annu. Rev. Pathol..

[27] Tomiyasu H., Habara M., Hanaki S., Sato Y., Miki Y., Shimada M. (2024). FOXO1 promotes cancer cell growth through MDM2-mediated p53 degradation. J. Biol. Chem..

[28] Jeong B., Park J.W., Kim J.G., Lee B.J. (2019). FOXO1 functions in the regulation of nicotinamide phosphoribosyltransferase (Nampt) expression. Biochem. Biophys. Res. Commun..

[29] Zhang H., Pan Y., Zheng L., Choe C., Lindgren B., Jensen E.D. (2011). FOXO1 inhibits Runx2 transcriptional activity and prostate cancer cell migration and invasion. Cancer Res..

[30] Wang H., Li Q.F., Chow H.Y., Choi S.C., Leung Y.C. (2020). Arginine deprivation inhibits pancreatic cancer cell migration, invasion and EMT via the down regulation of Snail, Slug, Twist, and MMP1/9. J. Physiol. Biochem..

[31] Xing Y.Q., Li A., Yang Y., Li X.X., Zhang L.N., Guo H.C. (2018). The regulation of FOXO1 and its role in disease progression. Life Sci..

[32] Hajaj E., Sciacovelli M., Frezza C., Erez A. (2021). The context-specific roles of urea cycle enzymes in tumorigenesis. Mol. Cell.

[33] Yousefi M., Nosrati R., Salmaninejad A., Dehghani S., Shahryari A., Saberi A. (2018). Organ-specific metastasis of breast cancer: molecular and cellular mechanisms underlying lung metastasis. Cell Oncol..

[34] Cabioglu N., Sahin A.A., Morandi P., Meric-Bernstam F., Islam R., Lin H.Y. (2009). Chemokine receptors in advanced breast cancer: differential expression in metastatic disease sites with diagnostic and therapeutic implications. Ann. Oncol..

[35] Mizuno R., Hojo H., Takahashi M., Kashio S., Enya S., Nakao M. (2022). Remote solid cancers rewire hepatic nitrogen metabolism via host nicotinamide-N-methyltransferase. Nat. Commun..

[36] Wang T., Rao D., Fu C., Sun Z., Luo Y., Lu J. (2025). MET promotes hepatocellular carcinoma development through the promotion of TRIB3-mediated FOXO1 degradation. Clin. Mol. Hepatol..

[37] Cui X., Zhao H., Wei S., Du Q., Dong K., Yan Y. (2023). Hepatocellular carcinoma-derived FOXO1 inhibits tumor progression by suppressing IL-6 secretion from macrophages. Neoplasia (New York, NY).

[38] Morris S.M. (2002). Regulation of enzymes of the urea cycle and arginine metabolism. Annu. Rev. Nutr..

[39] Gai X., Liu Y., Lan X., Chen L., Yuan T., Xu J. (2024). Oncogenic KRAS induces arginine auxotrophy and confers a therapeutic vulnerability to SLC7A1 inhibition in non-small cell lung cancer. Cancer Res..

[40] Jekabsons M.B., Merrell M., Skubiz A.G., Thornton N., Milasta S., Green D. (2023). Breast cancer cells that preferentially metastasize to lung or bone are more glycolytic, synthesize serine at greater rates, and consume less ATP and NADPH than parent MDA-MB-231 cells. Cancer Metab..

[41] Caldwell R.W., Rodriguez P.C., Toque H.A., Narayanan S.P., Caldwell R.B. (2018). Arginase: a multifaceted enzyme important in health and disease. Physiol. Rev..

[42] Nagasaka H., Tsukahara H., Yorifuji T., Miida T., Murayama K., Tsuruoka T. (2009). Evaluation of endogenous nitric oxide synthesis in congenital urea cycle enzyme defects. Metab. Clin. Exp..

[43] Kim J., Hu Z., Cai L., Li K., Choi E., Faubert B. (2017). CPS1 maintains pyrimidine pools and DNA synthesis in KRAS/LKB1-mutant lung cancer cells. Nature.

[44] Park H.J., Son H.J., Sul O.J., Suh J.H., Choi H.S. (2018). 4-Phenylbutyric acid protects against lipopolysaccharide-induced bone loss by modulating autophagy in osteoclasts. Biochem. Pharmacol..

