# Supplementary material for: FOXO1-mediated argininosuccinate lyase transcription inhibits ammonia metabolism and breast cancer cell metastasis
Source: J Biol Chem. 2025 Sep 2;301(10):110677. doi: 10.1016/j.jbc.2025.110677 (PMC12509978; doi:10.1016/j.jbc.2025.110677)
Supplement: Supplemental File [file mmc1.docx]

**FOXO1-** **mediated argininosuccinate lyase transcription inhibits ammonia metabolism and breast cancer cell metastasis**

Min Zhao^1, #^, Dongdong Yuan^1, #^, Mengmeng Wei^1, #^, Jie Zhang^1^, Wenjing Yang^2^, Shaojie Qin^2, *^, Le Li^1, *^

^1^ School of Life Sciences, Ningxia University, Yinchuan, China,750021

^2^ General Hospital of Ningxia Medical University, Yinchuan, China,750021

^#^ These authors are contributed equally

*Corresponding author: Le Li, [leli@nxu.edu.cn](mailto:leli@nxu.edu.cn); Shaojie Qin, [13629598315@163.com](mailto:13629598315@163.com)

Supplemental Information includes three figures and their figure legends.


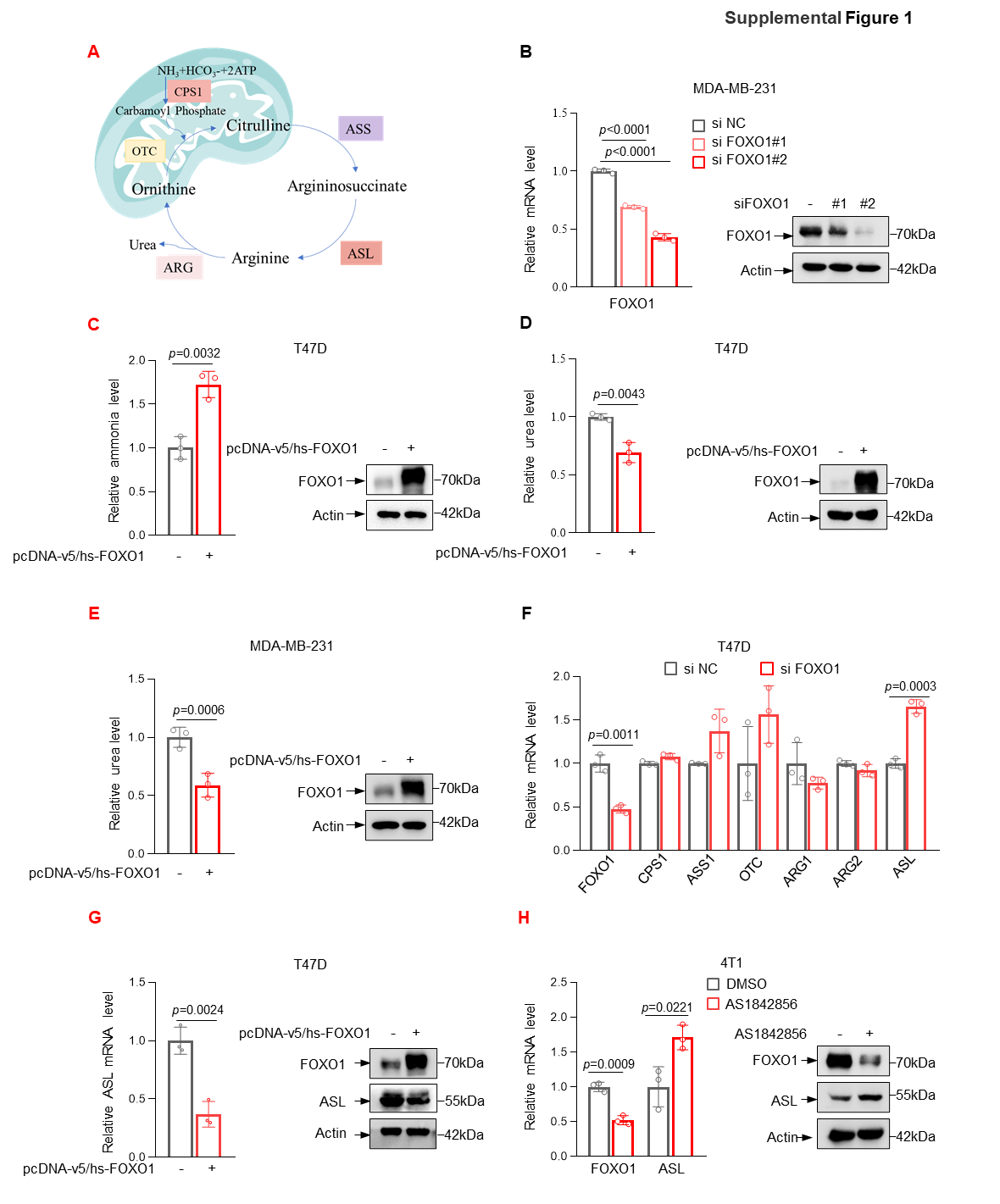


**Supplementary Figure 1. FOXO1 decreases ammonia clearance and controls the expression of the urea cycle enzyme ASL.**

A. Schematic illustration of the urea cycle and its regulatory role in breast cancer.

B. The knockdown efficiency of two FOXO1-specific siRNAs was analyzed by qRT-PCR and Western blotting. Actin was used as a loading control.

C-E. Relative levels of ammonia and urea following FOXO1 overexpression in MDA-MB-231 and T47D cells. Additionally, Western blot analysis was performed in MDA-MB-231 cells 48 hours after FOXO1 knockdown via siRNA. Actin was used as a loading control.

F. Effect of FOXO1 silencing on the expression of urea cycle enzymes in T47D cells.

G. The effect of FOXO1 overexpression on ASL expression in T47D cells was assessed by qRT-PCR and immunoblotting.

H. The effects of the FOXO1 inhibitor AS1842856 on ASL expression in 4T1 cells were analyzed by qRT-PCR and Western blotting.

Data are the mean ± SD. Each experiment was carried out at least 3 independent times. p values were calculated by 2-tailed unpaired Student’s t test. **p* < 0.05, ***p* < 0.01, ****p* < 0.001.


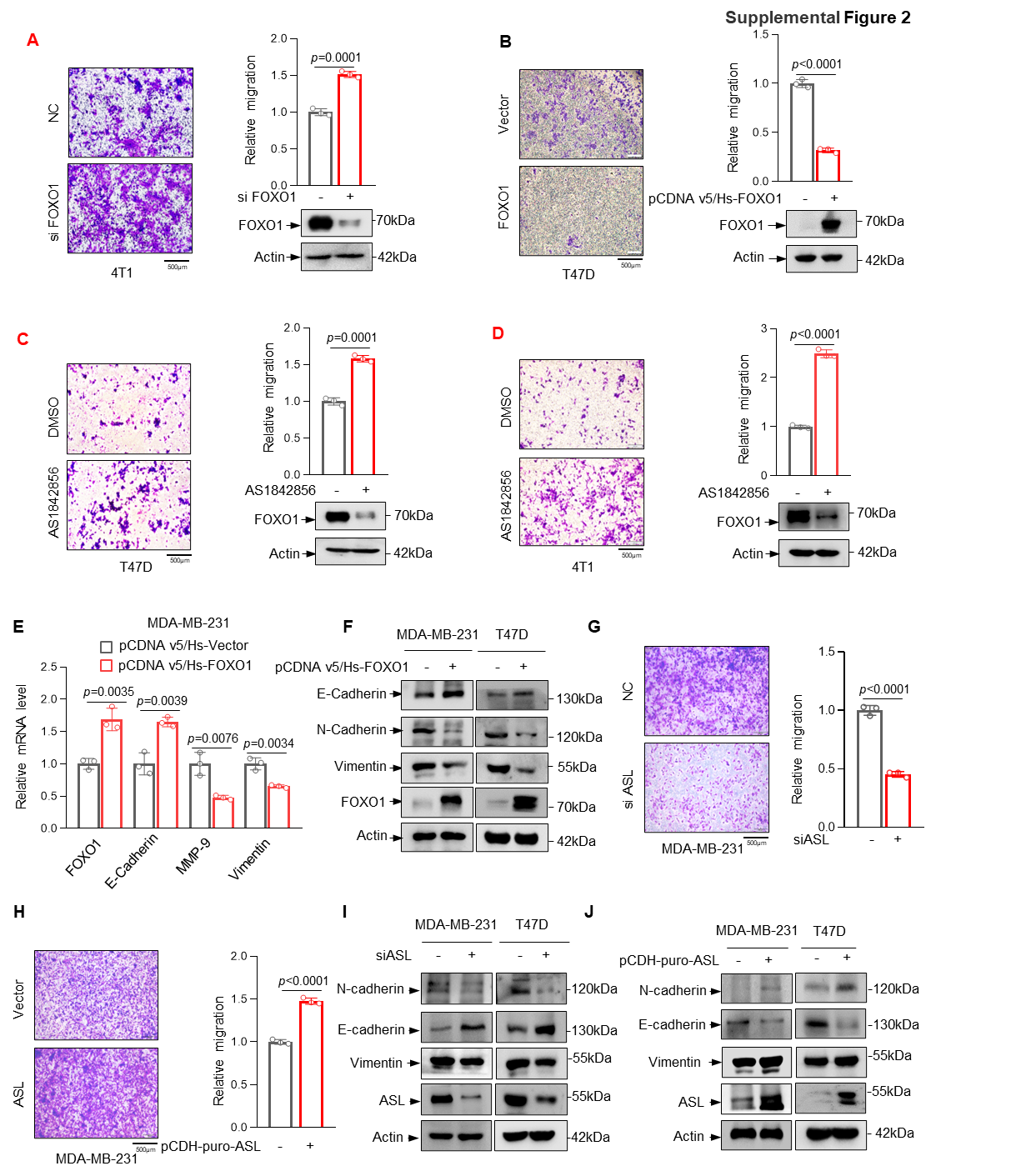


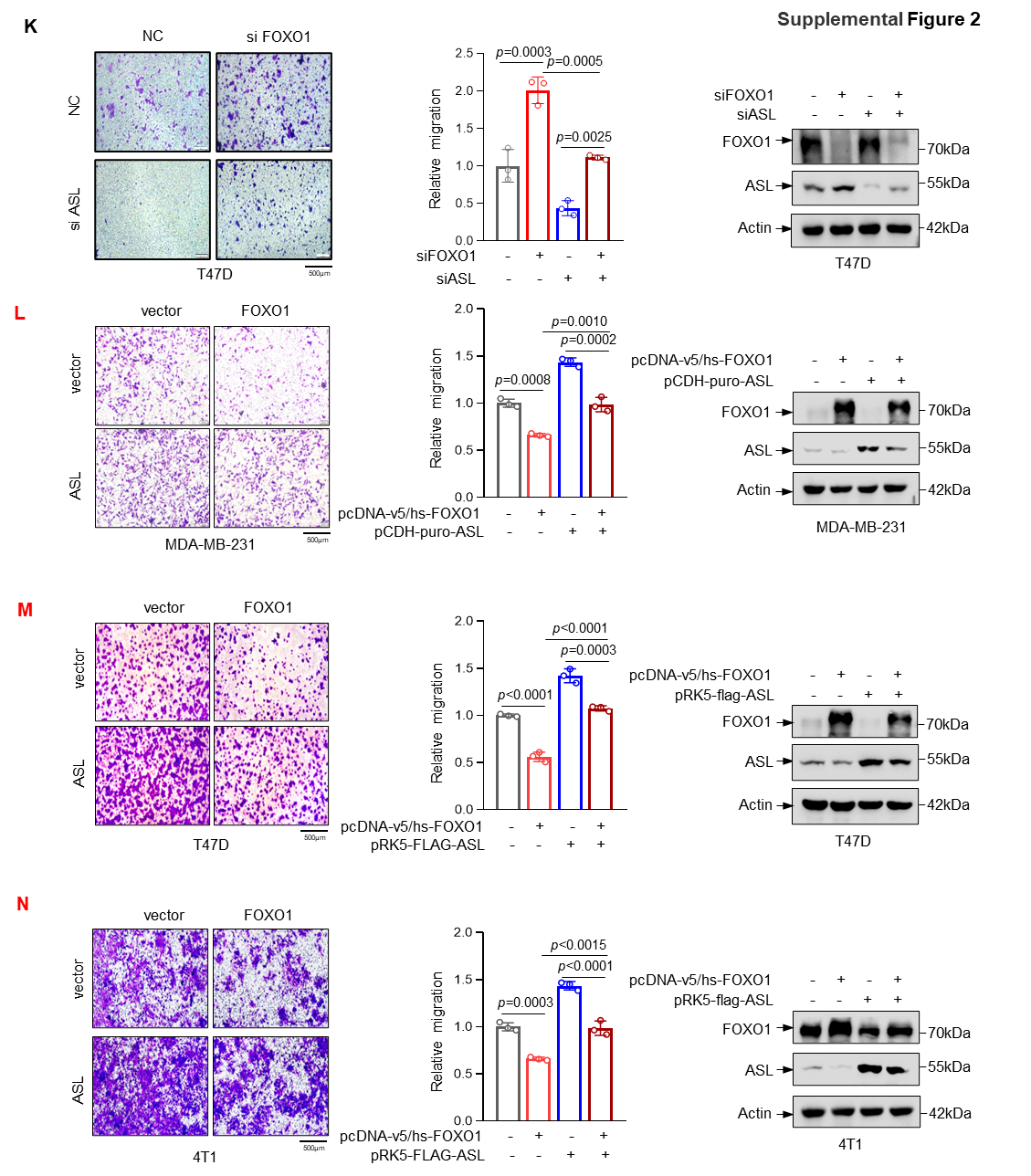


**Supplementary Figure 2. FOXO1 inhibits the migration of breast cancer cells by regulating ASL.**

A. The migratory ability of 4T1 cells with FOXO1 knockdown was assessed using a Transwell migration assay. Left: Representative image; Right: Quantification (cell counts) and Western blot analysis of FOXO1 expression. Scale bar: 500 μm. n = 3 independent experiments.

B. Transwell migration assay of T47D cells with FOXO1 overexpression. Representative images are shown on the left; quantitative data (analyzed by ImageJ) and Western blot results are shown on the right. Scale bar: 500 μm. n = 3.

C-D. Transwell migration assays of T47D and 4T1 cells treated with the FOXO1 inhibitor AS1842856. Representative images are shown on the left; quantitative data (analyzed by ImageJ) and Western blot results are shown on the right. Scale bar: 500 μm. n = 3.

E. FOXO1 was overexpressed in MDA-MB-231 cells, and the expression levels of EMT-associated proteins were quantified by qRT-PCR.

F. FOXO1 and EMT-associated protein levels were analyzed by immunoblotting.

G-H. Transwell migration assays were performed to assess the migratory ability of MDA-MB-231 cells following either ASL knockdown or overexpression. Representative images are shown on the left; quantitative data are presented on the right. Scale bar: 500 μm. n = 3.

I-J. ASL was knocked down or overexpressed in MDA-MB-231 and T47D cells. ASL and EMT-associated proteins were analyzed by immunoblotting.

K. Transwell migration assays were performed to evaluate the migration of T47D cells transfected with siRNAs targeting FOXO1, ASL, or both. Representative images are shown on the left; quantitative data are presented on the right. Scale bar: 500 μm. n = 3 independent experiments. Western blot analysis was conducted 48 hours post-transfection to assess FOXO1 and ASL protein levels. Actin was used as a loading control.

L-N. Transwell migration assays were performed to assess the migratory abilities of MDA-MB-231, T47D, and 4T1 cells after overexpression of FOXO1 or ASL. Representative images are shown on the left; quantitative data are presented on the right. Scale bar: 500 μm. n = 3. Western blotting was conducted 24 hours after overexpression to detect FOXO1 and ASL protein levels. Actin was used as a loading control.

Data are the mean ± SD. Each experiment was carried out at least 3 independent times. p values were calculated by 2-tailed unpaired Student’s t test or two-way ANOVA followed by Tukey’s multiple-comparison test. **p* < 0.05, ***p* < 0.01, ****p* < 0.001.


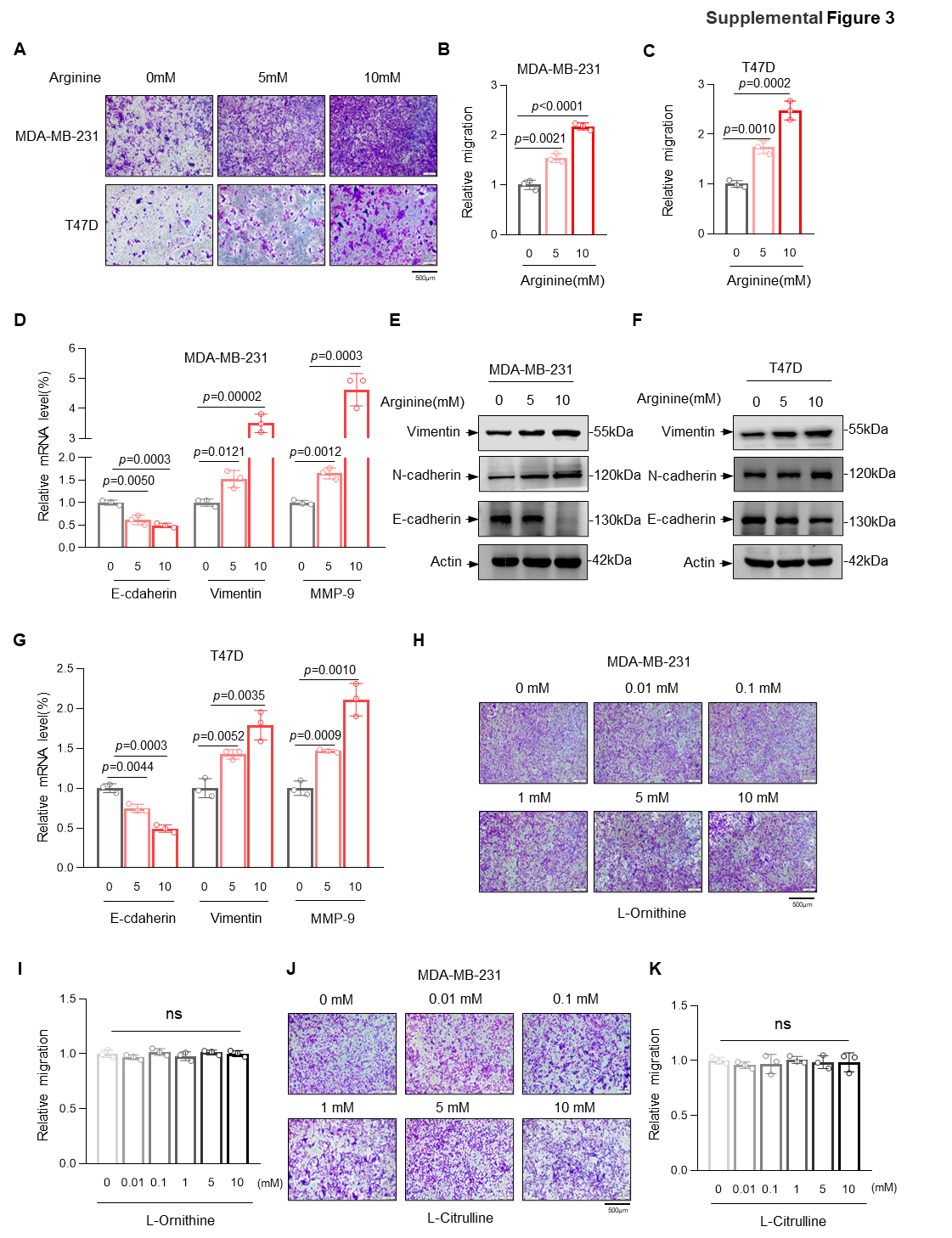


**Supplementary Figure 3. ASL promotes breast cancer cell migration by regulating arginine.**

A-C. Transwell migration assays were performed to assess the migration capacity of MDA-MB-231 and T47D cells supplemented with arginine. Representative images are shown on the left, and quantitative data are presented on the right. Scale bar: 500 μm; n = 3 independent experiments.

D-G. Arginine supplementation in MDA-MB-231 and T47D cells. EMT-associated protein expression was analyzed by qRT-PCR and immunoblotting.

H-K. Transwell migration assays were performed to assess the migration capacity of MDA-MB-231 cells supplemented with L-ornithine or L-citrulline. Representative images are shown above, and quantitative data are presented below. Scale bar: 500 μm; n = 3 independent experiments.

Data are the mean ± SD. Each experiment was carried out at least 3 independent times. p values were calculated by 2-tailed unpaired Student’s t test. **p* < 0.05, ***p* < 0.01, ****p* < 0.001.
